# Genome-wide association mapping in hairy vetch (*Vicia villosa*) discovers a large effect locus controlling seed dormancy

**DOI:** 10.3389/fpls.2023.1282187

**Published:** 2023-10-24

**Authors:** Neal Tilhou, Lisa Kissing Kucek, Brandon Carr, Annie Marion, Joel Douglas, John Englert, Shahjahan Ali, John Raasch, Suresh Bhamidimarri, Steven Brian Mirsky, Maria J. Monteros, Sarah Krogman, Ryan Hayes, Mark Azevedo, Heathcliffe Riday

**Affiliations:** ^1^ US Dairy Forage Research Center, United States Department of Agriculture-Agricultural Research Service (USDA-ARS), Madison, WI, United States; ^2^ James E. “Bud” Smith Plant Materials Center, United States Department of Agriculture-Natural Resources Conservation Service (USDA-NRCS), Knox City, TX, United States; ^3^ Corvallis Plant Materials Center, USDA-NRCS, Corvallis, OR, United States; ^4^ Central National Technology Support Center, USDA-NRCS, Fort Worth, TX, United States; ^5^ National Plant Materials Program, USDA-NRCS, Washington, DC, United States; ^6^ Corteva Agriscience, Connell, WA, United States; ^7^ Sustainable Agricultural Systems Laboratory, United States Department of Agriculture-Natural Resources Conservation Service (USDA-ARS), Beltsville, MD, United States; ^8^ Bayer Crop Science, Chesterfield, MO, United States; ^9^ School of Medicine in Kansas, University of Kansas Medical Center, Wichita, KS, United States; ^10^ Forage Seed and Cereal Research Unit, USDA-ARS, Corvaillis, OR, United States

**Keywords:** seed dormancy, genomewide association, cover crop, legume, genomics

## Abstract

Hairy vetch (*Vicia villosa* Roth), a winter-hardy annual legume, is a promising cover crop. To fully leverage its potential, seed production and field performance of *V. villosa* must be improved to facilitate producer adoption. Two classic domestication traits, seed dormancy (hard seed) and dehiscence (pod shatter), are selection targets in an ongoing breeding program. This study reports a genome-wide association study of 1,019 *V. villosa* individuals evaluated at two sites (Knox City, Texas and Corvallis, Oregon) for the proportion of dormant seed, visual pod dehiscence scores, and two dehiscence surrogate measures (force to dehiscence and pod spiraling score). Trait performance varied between sites, but reliability (related to heritability) across sites was strong (dormant seed proportion: 0.68; dehiscence score: 0.61; spiraling score: 0.42; force to dehiscence: 0.41). A major locus controlling seed dormancy was found (*q*-value: 1.29 × 10^−5^; chromosome 1: position: 63611165), which can be used by breeding programs to rapidly reduce dormancy in breeding populations. No significant dehiscence score QTL was found, primarily due to the high dehiscence rates in Corvallis, Oregon. Since Oregon is a potentially major *V. villosa* seed production region, further dehiscence resistance screening is necessary

## Introduction

Cover crop adoption can improve farm productivity and reduce environmental harm (Poeplau and Don, 2015; Tiemann et al., 2015; Austin et al., 2017). Cover crops can reduce nutrient loss (Wyland, 1996; Drinkwater et al., 1998), improve soil health (Sainju and Singh, 1997), and decrease yield loss due to pests and weeds (Teasdale, 1996). A total of 6.2 million hectares of cover crops were planted in 2017 (USDA-NASS, ; Wallander et al., 2021), an increase of 50% from 2012. This indicates that 5.1% of US-harvested, non-alfalfa cropland used cover crops in 2017. Further cover crop adoption requires improved agronomic methods and breeding improved varieties in a wider suite of cover crop plant species (Kaspar and Bakker, 2015; Wallace et al., 2017; Wayman et al., 2017).

As an agricultural method, cover cropping is a broad term with goals varying by environmental constraints and producer needs. Currently, small grains (cereal rye (*Secale cereale* L.), wheat (*Triticum aestivum* L.), oats (*Avena sativa* L.), or barley (*Hordeum vulgare* L.)) collectively account for >90% of cover crop acreage (Wallander et al., 2021; note this survey did not ask about cover crop mixes). Small grains are affordable, already familiar to farmers, and established rapidly. Cereal rye is popular because it can overwinter in the northern USA and produces abundant biomass, which can provide early-season weed control (Wallace et al., 2017). However, there is concern that small grain residues break down too slowly and limit nitrogen availability for the following cash crop (Wagger et al., 1998; Wallace et al., 2017; Nevins et al., 2020; Thompson et al., 2020). The extent of this issue likely varies by location and cropping system (Snapp and Surapur, 2018; Bourgeois et al., 2022).

The inclusion of legume species (family Fabaceae) in fall-planted cover crop mixes allows atmospheric nitrogen fixation. Further, legume residue decomposes more rapidly than grass residue due to lower carbon-to-nitrogen ratios. Therefore, legumes in fall cover crop plantings will improve the nutrient availability and yield of the following cash crops (Ranells and Wagger, 1996; Clark et al., 1997; Vyn et al., 2000; Singh et al., 2020). Hairy vetch is a commonly grown legume cover crop due to its winter hardiness, high biomass yields, and nitrogen supply to the subsequent cash crop (Power and Zachariassen, 1993; Brandsæter et al., 2002; Parr et al., 2011; Mirsky et al., 2017). *V. villosa* is a diploid (2*n* = 14, Kahlaoui, 2009) allogamous species (Zhang and Mosjidis, 1995) that tolerates some self-pollination (Hamadi et al., 2012). Various bee species, including *Bombus* sp. and *Apis mellifera*, facilitate cross-pollination.

Hairy vetch has limited adoption in current agricultural systems due to its incomplete domestication (Wayman et al., 2017). *V. villosa* produces dormant seed (i.e., hard seed) and is prone to early shatter (pod dehiscence). Both traits are typically eliminated during crop domestication (Doebley et al., 2006; Purugganan and Fuller, 2009; Bohra et al., 2022). Due to limited historic and current investment in forage and cover crop breeding, seed dormancy and pod dehiscence persist in *V. villosa* cultivars (Wilke and Snapp, 2008). Like many members of the Faboideae family (Smýkal, 2014), physical seed dormancy in *V. villosa* is linked to a water-impermeable seed coat that prevents imbibition of water (Jones, 1928; Renzi et al., 2014). Prior reports of *V. villosa* seed dormancy rates range from% 0 to 100% (Jannink, 1997; Jacobsen et al., 2010; Renzi et al., 2014; Mirsky et al., 2015; Kissing Kucek et al., 2020a). Dormant seed of *V. villosa* persistent in the soil seed bank leads to weedy *V. villosa* plants, which can reduce cash crop yields (Curran, 2015; Mirsky et al., 2015). In addition, *V. villosa* and small grain seeds mature at the same time, therefore requiring the use of expensive and specialized threshing equipment to separate small grain seed harvests to separate volunteer *V. villosa* seed from small grain seed.

Pod shatter, also known as dehiscence, contributes to seed yield losses and can create weedy “volunteer” *V. villosa* populations (Kissing Kucek et al., 2020a). One study reported 3% to 60% shattered pods among evaluated *V. villosa* lines (Abd El-Moneim, 1993). For seed growers, *V. villosa*’s propensity for pod dehiscence, in combination with the crop’s indeterminant growth habit, makes decisions on harvest timing challenging. The result can be extensive seed yield losses. The reduced seed harvestability due to pod shattering results in expensive *V. villosa* seed that can become a barrier for farmers looking for an inexpensive legume cover crop seed (Renzi et al., 2023).

Using recurrent phenotypic selection, plant breeders should be able to gradually reduce seed dormancy and pod dehiscence after repeated selection cycles. Opportunities to integrate DNA markers linked to these traits could accelerate this breeding progress. Research in previously domesticated legume crops indicates that seed dormancy and pod dehiscence can be eliminated by a small number of large effect loci, and consequently, these traits are excellent candidates for marker-assisted selection. For example, seed dormancy was eliminated by a single altered gene in *Lens culinaris* Medic. (Ladizinsky, 1985), *Lupinus angustifolius* L. (Forbes and Wells, 1968), *Vigna unguiculata* L. (Kongjaimun et al., 2012), *V. umbellata* (Thunb.) (Isemura et al., 2010), and *V. radiata* L. (Isemura et al., 2012). In *Pisum sativum* L., two to three loci eliminated seed dormancy (Weeden, 2007). A single recessive allele removed physiological dormancy in *Medicago truncatula* Gaertn. (Chai et al., 2016). In soybean (*Glycine max* (L.) Merr.), a recessive single-point mutation of *Gmhs1-1* created soft seed by modifying a calcium-based seed coat membrane protein (Sun et al., 2015). Alternative mutations at *qHS1 in G. max* also formed cracks in the seed coat that caused partial imbibition (Jang et al., 2015; Sun et al., 2015).

Similar to seed dormancy, pod dehiscence can be controlled by large-effect loci. Ladizinsky (1985) reported one to two dominant genes in *Lens* species. Two genes controlled dehiscence in *Pisum satium* L. (Weeden, 2007). Abd El-Moneim (1993) found that nonshattering in *V. sativa* L. could be eliminated through one large effect recessive gene. In *V. radiata* (L.) Wilczek), one quantitative trait loci (QTL) was associated with pod dehiscence (Isemura et al., 2012).

Potential large effect loci for domestication traits could be obscured by environmental effects such as relative humidity (Quinlivan, 1968) and temperature at seed maturity (Kissing Kucek et al., 2020a), pod maturity at harvest (Samarah, 2006), and pod handling. Postharvest seed storage can also alter dormancy in *V. villosa* (Renzi et al., 2014). Such mechanisms are common in winter annual species of Mediterranean origin since hot summer weather signals upcoming wet and cool fall conditions that are ideal for germination (Williams and Elliott, 1960). Within the palisade layers of the seed coat, lipids and other hydrophobic compounds degrade in high heat and allow germination (Smýkal et al., 2014).

The objective of this study is to determine trait genetic associations for pod dehiscence and seed dormancy in a diverse set of *V. villosa* germplasm from an existing breeding program. This population varies for both seed dormancy and pod dehiscence measurements (Kissing Kucek et al., 2020b, 2020a). These observations and the presence of large effect loci in other legumes (see above) highlight the promise of marker-assisted selection to accelerate *V. villosa* development. This study reports (1) the presence of significant QTL in *V. villosa* breeding populations and (2) estimates of the genetic reliability and phenotypic correlations among seed dormancy, dehiscence, and other surrogate traits.

## Materials and methods

### Germplasm and experimental design

In 2015 and 2016, researchers in the Cover Crop Breeding Network (www.covercropbreeding.com) performed a search of available local and internationally collected germplasm with the goal of finding germplasm with traits beneficial for cover crops. The team selected and acquired 27 V*. villosa* populations for the breeding program, including breeding material developed in Maryland, Minnesota, North Carolina, and Wisconsin. The ancestry and location of origin of these 27 populations are described in Kucek et al. (2019). In the fall of 2016, 12,500 *V. villosa* genotypes from the 27 populations were evaluated at six sites (Rocky Mount, North Carolina; Clayton, North Carolina; Beltsville, Maryland; Ithaca, New York; Prairie du Sac, Wisconsin; and Roseville, Minnesota). Each site assessed emergence, fall and spring vigor, winter survival, and flowering time, and two sites assessed biological nitrogen fixation (Kucek et al., 2019). The top 3% to 6% of individuals at each site were allowed to flower and contribute to crossing blocks.

The current experiment used 80 maternal half-sibling lines derived from selections from 24 of the 27 initially selected populations (**Table 1**). The 80 parents were selected based on divergent variations in seed dormancy and pod dehiscence traits. Individual open-pollinated progeny from these half-sibling lines were assigned to one of two sites (Oregon and Texas; 10 progeny per maternal line per site). The Oregon site was planted at the USDA-Natural Resources Conservation Service (NRCS), Corvallis Plant Materials Center in Oregon (OR) on 25 September 2018, in a Woodburn silt-loam (fine-silty, mixed, superactive, mesic Aquultic Argixerolls), and the Texas site was planted at the USDA-NRCS, James E. “Bud” Smith Plant Materials Center in Knox City (TX) on 16th October 2018 in an Altus fine sandy-loam (fine-loamy, mixed, superactive, thermic Pachic Argiustolls). Individual unreplicated progeny were randomly assigned to rows within 10 balanced blocks of half-sibling families within each location, with 1.83 m between individuals and 1.83 m between rows. Field traits were measured in 2019 at both sites. The Oregon site experienced almost complete fall predation, likely from slugs (Gastropods), and therefore transplants were established into landscape fabric in late winter. Texas relied on manual rouging to remove weeds. To control for bruchid beetle (*Bruchus brachialis* Fahraeus) damage, Zeta-cypermethrin (Mustang Maxx; 0.0028 kg A.I. ha^−1^) was applied in 2019 in Oregon on June, 2 July, 11 July, 21 and July and in Texas on 10 May, 17 May, 25 May, and 31May 31.

**Table 1:** The number (*n*) of families and individuals evaluated for seed dormancy and dehiscence based on initial germplasm origin.

Population	*n* Families	*n* Individuals	Notes
Albert Lea Organic	5	93	2016 VNS (US)
Minnesota Buckwheat Growers	1	17	2016 VNS (US)
MSP4045	7	120	University of Minnesota Breeding Population
MSP4046	8	137	University of Minnesota Breeding Population
MSP4047	3	48	University of Minnesota Breeding Population
MSP4048	5	94	University of Minnesota Breeding Population
MSP4049	6	101	University of Minnesota Breeding Population
North Dakota Podoll	1	20	Population from David Podoll (US)
Nebraska	2	31	
PI 263190	6	103	U.S. National Plant Germplasm System
PI 268321	2	34	U.S. National Plant Germplasm System
PI 491408	1	16	U.S. National Plant Germplasm System
PI 536642	2	29	U.S. National Plant Germplasm System
Purple Bounty	2	37	Cultivar (US, Moore et al., 2020)
Purple Prosperity	3	48	Cultivar (US, Moore et al., 2020)
Savane	1	14	Cultivar (France)
VIC051	4	59	Serbian breeding population
VIC410	4	58	Serbian breeding population
WIHV1	2	35	USDA-ARS breeding population
WIHV2	2	37	USDA-ARS breeding population
WIHV3	4	54	USDA-ARS breeding population
WIHV6	2	33	USDA-ARS breeding population
WIHV7	4	66	USDA-ARS breeding population
WIHV8	3	48	USDA-ARS breeding population

VNS, variety not stated.

### Data collection

Maturity ratings based on Kalu and Fick (1981) were performed when 50% of individuals had flowers at the Texas location and when 50% of individuals had pods at both sites in 2019. These maturity ratings were collected due to early maturity being a potential breeding target, and plant maturity could serve as a covariate for pod dehiscence and seed dormancy.

Between early June and mid-August of 2019, pods developed a brown color, a signal of physiological maturity. Around 50 pods were collected from each individual at each site. If pods appeared to contain few seeds per pod, additional pods were collected per plant. Pods were phenotyped for traits related to pod dehiscence and seed dormancy at the USDA-ARS Dairy Forage Research Center in Madison, WI in 2020. Detailed methods of visual dehiscence ratings and seed dormancy determination are described in (Kissing Kucek et al., 2020b) and Kissing Kucek et al. (2020a), respectively. Briefly, all pods collected from an individual plant received a visual pod dehiscence score, targeting 50 pods per individual. Pods were dried at 28°C for 24 h before evaluation. Dehiscence was scored on a zero to three scale, with zero indicating a fully intact pod (no openings along sutures), one indicating one suture was partially opened (one side of the pod), two indicating two sutures were partially opened (both sides of the pod), two and a half indicating a pod was partially winged apart, and three indicating the pod had fully opened. Individuals with fewer than 10 pods rated were not included in subsequent data analysis. Individual plants with flat pods from lack of seed fill were also removed. Individual plants with >66% immature pods were removed from dehiscence measurements.

Each seed from an individual received a binomial rating for seed dormancy: “0” if the seed imbibed water and produced a radicle without scarification, or “1” if the seed did not imbibe water after 7 days and produced a radicle after scarification. Nonviable seeds or seed-imbibed water that did not produce a radicle were removed from subsequent analysis. Individuals who did not have at least 10 viable seeds observed were also removed from further analysis.

Pod samples were rated for pod spiraling on a visual scale of zero, one, and two (further details are reported in Kissing Kucek et al., 2020b). For individuals with >66% immature pods, spiraling values were set to not applicable (NA), due to a relationship between immaturity and spiraling.

Individual plants with at least three fully closed pods were screened for force to dehiscence using an MTS Insight^®^ 1kN (MTS Systems Corporation) instrument for measuring cracking force on unopened pods. The evaluation used a 100-N load cell and TestWorks ^®^ [v.4.11B] (MTS Systems Corporation: Eden Prairie, MN, USA) set to 96% break sensitivity, 0.1 N break threshold, and 25 Hz data acquisition rate. Individual plants with fewer than three pods tested for force to dehiscence were excluded from the analysis. Samples with immature green pods, or pods that were flat and did not fill with seeds were removed from analysis.

### Hairy vetch dormancy and shatter calculation of BLUPs

Broadly, models were used to partition variance and remove covariates prior to genome-wide association studies (GWAS) analysis. The analysis did not use models with complex genotype by environment interactions between the sites since any site-specific QTL will not be useful for the breeding program. For pod dehiscence, the best linear unbiased predictors (BLUPs) of each plant were extracted using a multinomial logistic regression model implemented through ASReml-R (Butler et al., 2017), with 1,019 individuals representing all 80 maternal lines that met inclusion criteria for analysis for visual dehiscence. Visual pod dehiscence BLUPs were also generated separately by site, as the highly skewed Oregon data violated model assumptions.


(1) $$f\left(\text{k},\text{n}\right)={\text{β}}_{\text{k}}\cdot {\text{χ}}_{\text{n}}$$

Where *f*(*k*,*n*) indicates the cumulative probability of each pod observation in taking a value of the *k*th category of dehiscence, where *k* = 0, 1, 2, 2.5, 3, and *χ_n_* is the set of explanatory variables including fixed effect maternal line i and the random effect of site j.

A total of 713 individual plants, representing all 80 maternal lines, met inclusion criteria for force-to-dehiscence analysis. Values for force to dehiscence (*N*) were right skewed and consequently square root transformed to create a normally distributed set of observations. BLUPs for each plant were obtained using Eq. (2):


(2) $${\text{Y}}_{\text{ijkl}}={\text{α}}_{\text{i}}+{\text{β}}_{\text{j}}+{\text{δ}}_{\text{k}}+{\text{γ}}_{\text{l}}+\text{ϵ}$$

Where *Y*_ijkl_ indicates the square root of force (*N*) required to open a closed pod from the fixed effect *α* of maternal line i, the random effect *β* of site j, the random effect *δ* of individual plant k, the random covariate *γ* of pod maturity rating, and residual error *ϵ*. BLUPs for each individual plant were calculated by adding random coefficients from the *β*_j_ term to the fixed coefficients from the *α*_i_ term.

For seed dormancy, 1,019 individual plants, representing all 80 maternal lines, met inclusion criteria for analysis. BLUPs for seed dormancy were extracted as the coefficient of individual plant j added to the coefficient of maternal line i from Eq. (3) in ASReml-R:


(3) $${\text{Y}}_{\text{ijklm}}={\text{β}}_{0}+{\text{β}}_{1}{\text{x}}_{\text{i}1}+{\text{β}}_{2}{\text{x}}_{\text{j}2}+{\text{β}}_{3}{\text{x}}_{\text{k}3}$$

Where *Y*_ijklm_ is the log-likelihood of dormant seed (0,1) for maternal line i, individual plant j, site k, technical replicate l, and maturity covariate m; *B*_0_ is the intercept of log odds, *β*_1_ and *x*_i1_ are the partial slope and fixed variable associated with maternal line i, *β*_2_ and *x*_j2_ are the partial slope and random variable associated with individual plant j, *β*_3_ and *x*_k3_ are the partial slope and random variable associated with site k, *β*_4_ and *x*_l4_ are the partial slope and random variable associated with technical replicate l, *β*_5_ and *x*_m5_ are the partial slope and random variable associated with the pod maturity covariate m.

Spiraling data were not corrected through analysis due to only one observation per individual plant. However, it was included in the GWAS analysis after adjusting for site means.

### Tissue sampling, sequencing, bioinformatics, and genome-wide association

*V. villosa* tissues were collected for sequencing during active vegetative growth. At the Oregon location, actively expanding penultimate leaves were sampled just before and during early bloom on three separate dates (19 May, 7 June, and 14 June), and the Texas location plants were sampled on 14 April. Each field sample was placed into a labeled coin envelope and immediately placed on ice before transport to a laboratory freezer (−20°C). The samples were subsequently frozen to −60°C and lyophilized prior to shipment. Twice. Once at the University of Wisconsin Biotechnology Center and once at Oklahoma State University on an Illumina sequencer (NovaSeq 6000). The University of Wisconsin used an *NsiI-BfaI* double digestion restriction enzyme digestion and Oklahoma State University Oklahoma State University used an *ApeKI* restriction enzyme digestion. Fragments were then ligated to barcoded adaptors prior to polymerase chain reaction amplification.

**Figure 1:**
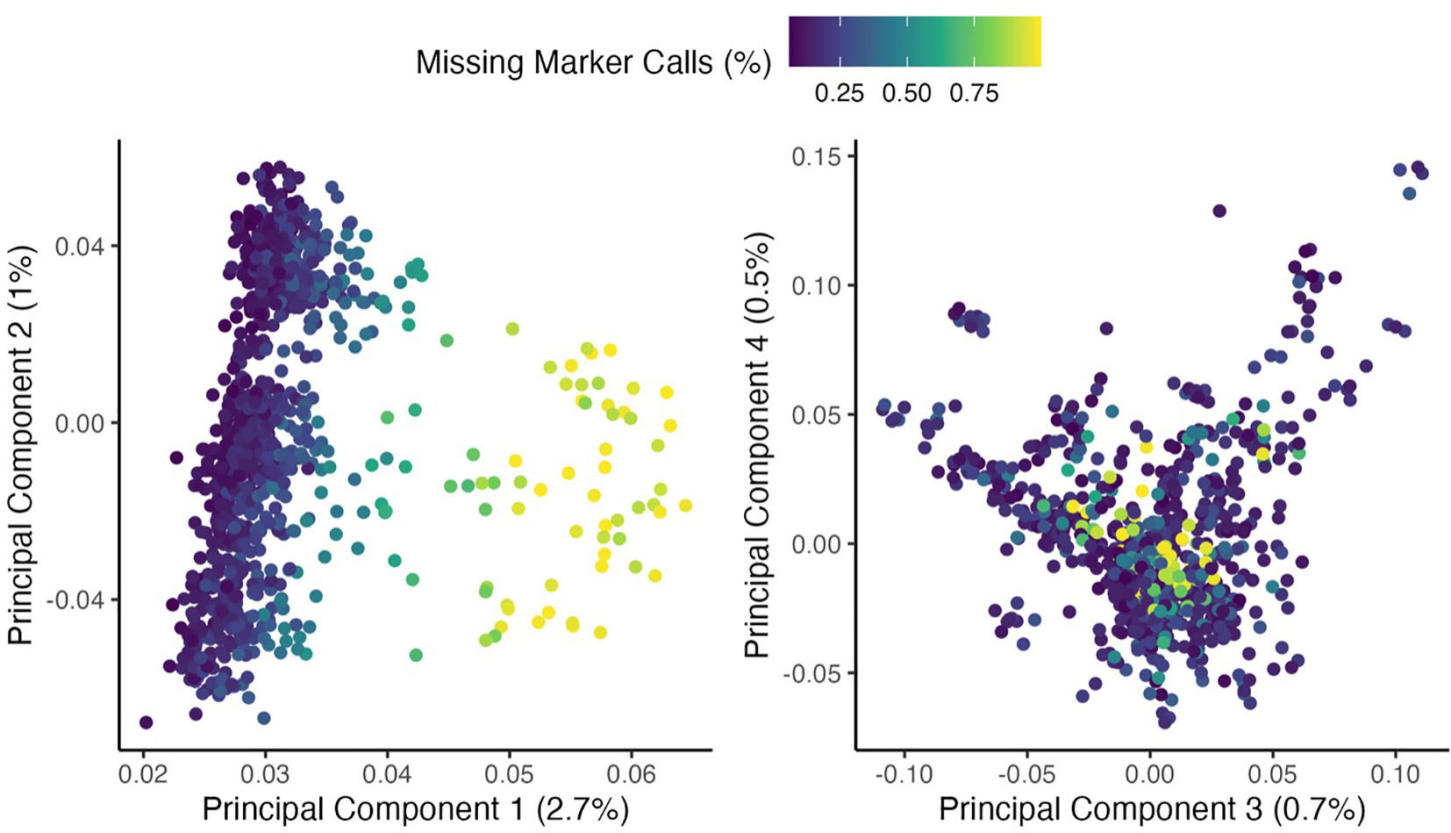
The first four principal components of 956 V*. villosa* genotypes are based on 100,000 randomly selected SNPs. Point colors indicate the proportion of missing markers in each individual prior to imputation. The percent variance of each principal component is indicated in parentheses. Individuals with<1 mean read depth per site were removed from further analysis (population mean: 4.16). This figure does not contain an out-group (out-group presented in **Supplementary Figure S1**).

Bioinformatics sequence data processing was completed at the University of Wisconsin Biotechnology Center, which merged the two sequencing runs. Sequencing output analysis used the TASSEL analysis platform (Glaubitz et al., 2014). Barcoded sequence read outputs were collapsed into a set of unique sequence tags with counts. These tags were aligned to the reference genome (*V. villosa* v1.1; Fuller et al., 2023). Each tag was assigned to a position with the best unique alignment, and the occupancies of tags for each sample were observed from barcode data. Overall, 2,524,985 single-nucleotide polymorphism markers (SNPs) were present prior to filtering. A subset (*n* = 74) of individuals had a means<1 reads per site, relative to the mean of 4.16 reads per site (SD = 1.28) in the overall population (**Figure 1**). Over 90% of these samples were collected from the Texas location and were not stratified by genetic background; therefore, this was presumed to be a leaf tissue storage issue and these individuals were removed from further analysis. The 38% of markers containing<0.5 read per site and the 26% of SNPs that mapped outside the seven primary chromosomes in the assembly were removed. A random subset of 100,000 SNP markers was used to evaluate inter-relationships within the dataset using principal component analysis. This revealed a strong out-group, which was omitted from further analysis (*n* = 137; discussed briefly below). Finally, markers with minor allele frequencies of<0.025 were removed. In total, 1,010,403 SNP markers across 881 individuals met the filtering criteria. The imputation of missing markers (42%) was done through the Beagle program (Browning et al., 2018).

Significant QTLs were determined using the GWAS function in the R package “sommer” v.4.3.1 (Covarrubias-Pazaran, 2016). The analysis included force-to-dehiscence BLUPs (*n* = 500), pod dehiscence BLUPs (*n* = 791), seed dormancy BLUPs (*n* = 853), and pod spiraling scores (*n* = 797). Population structure was accounted for by including the realized relationship matrix (K model), and the interactive effect of each maternal line at each site was included as a random effect. The GWAS model also allowed different error variances between sites (diagonal residual covariance structure). Multiple-testing correction was based on the false discovery rate (Benjamini and Hochberg, 1995) with a significance threshold of *q* = 0.01. Reliability, a measure related to heritability on an entry-difference basis, was calculated for each trait using the genotypic variance and error variance estimated within the GWAS model (Schmidt et al., 2019).

## Results

### Field observations and BLUP calculations

Maturity ratings at 50% flowering were not collected at the Oregon site, but maturity ratings at 50% pod development had positive (0.51) correlations between the Texas and Oregon sites. Maturity covariates did not correlate strongly with the target traits and only improved model fit for force to dehiscence during BLUP calculation (**Supplementary Figure S2**). Maturity covariates were not included in the models for visual shatter, spiraling, or seed dormancy. Trait correlations generally were low after accounting for differences in site means (**Supplementary Figure S3**). The only exception was the correlations (*p*< 0.001) between pod dehiscence scores and pod spiraling scores (Oregon: 0.37, Texas: 0.58), and seed dormancy and pod dehiscence scores (Oregon: 0.26, Texas: 0.32).

Mean and trait distributions of visual pod dehiscence were heavily influenced by the test site (mean Oregon: 2.70, SD: 0.53; mean Texas: 0.90, SD: 0.67; **Supplementary Figure S3**). The Oregon site had few observations in low pod–dehiscence classes, resulting in a strong left skew (**Supplementary Figure S2**). Within the Texas site, maternal line, individual plant, and error contributed similar variance to visual dehiscence (**Table 2**). The reliability of the pod dehiscence score was 0.61, but the correlation between mean site family performance was 0.21.

**Table 2:** Variance components with standard errors in parentheses for the models of pod shatter score (Eq. 1), force to shatter (Eq. 2), and hard seed (Eq. 3).

Trait	Site	Maternal line	Individual plant	Residual	Reliability	Between site-year correlation (family means)
Dehiscence score	–^a^	0.91 (0.16)	0.94 (0.07)	1	0.61	0.21
Force to shatter (peak *N*)	0.001 (0.002)	0.009 (0.003)	0.060 (0.005)	0.178 (0.004)	0.41	0.36
Dormancy proportion	0.34 (0.51)	0.58 (0.12)	1.98 (0.09)^b^	1	0.68	0.51
Spiraling score	–	–	–	–	0.42	0.19

Reliability was calculated on an entry-difference basis and reported between site and year correlations based on family means of raw phenotypic values between sites.

a The Oregon site was omitted due to an insufficient number of low dehiscence observations.

b Variance of technical replicates for hard seed proportion was 0.19 (0.19).

Raw force to dehiscence (*N*) scores had only mild right skew distributions (**Supplementary Figure S2**). The variance of force to dehiscence was dominated by the influence of residual error among pods within an individual plant, followed by an individual plant, followed by the maternal line, and then the site (**Table 2**). Force to dehiscence was only measured on the low-shatter individuals (mean visual dehiscence scores: 1.1 with force to dehiscence measurements vs. 2.8 without force-to-dehiscence measurements, *p*< 0.001). The site had a very small influence on force to dehiscence (mean Oregon: 2.38, SD: 1.29; mean Texas: 2.15, SD: 0.95; **Table 2**). Force-to-shatter reliability was 0.41, and correlation between site and mean family performance correlation was 0.36.

The mean seed dormancy was 58%, with a large number of individuals with >75% dormancy and some individuals with zero measured dormant seed (**Supplementary Figure S2**). Seed dormancy variance was dominated by the influence of individual plants, followed by residual error, then maternal line, site, and technical replicates (**Table 2**). The site had a small influence on seed dormancy means (mean Oregon: 0.66, SD: 0.28; mean Texas: 0.52, SD: 0.27; **Table 2**). Despite only one observation per genotype, pod spiraling had a reliability of 0.42 and between site and mean family correlation of 0.19. Similar to dehiscence, there were fewer observations of low pod spiraling rankings in the Oregon site (mean Oregon: 1.92, SD: 0.33; mean Texas: 1.03, SD: 0.82; **Table 2**).

### Genetic relationships and GWAS analysis

Principal component analysis revealed the presence of two major subpopulations in the breeding program (**Supplementary Figure S1**). Germplasm in the outgroup is consistently traced back to a small number of germplasm sources (Nebraska, PI 268321, ‘Purple Bounty’, and ‘Purple Prosperity’). These individuals appear to be distantly related to the core *V. villosa* populations based on their poor alignment with the *V. villosa* genome assembly, and they also appear to not cross-pollinate with the core breeding population (**Supplementary Figure S1**).

Broadly, quantile–quantile plots indicated that the inclusion of the K matrix and half-sibling family effect controlled potential *p*-value inflation in the GWAS (**Supplementary Figure S4**). The GWAS panel included individuals from a relatively small number of half-sibling families (*n* = 80). This had the potential to introduce population structure and inflate marker effect estimates. The solution for this was to include a covariate to remove the maternal effect, which resulted in the removal of a large portion of informative genetic variance from the GWAS.

**Figure 2:**
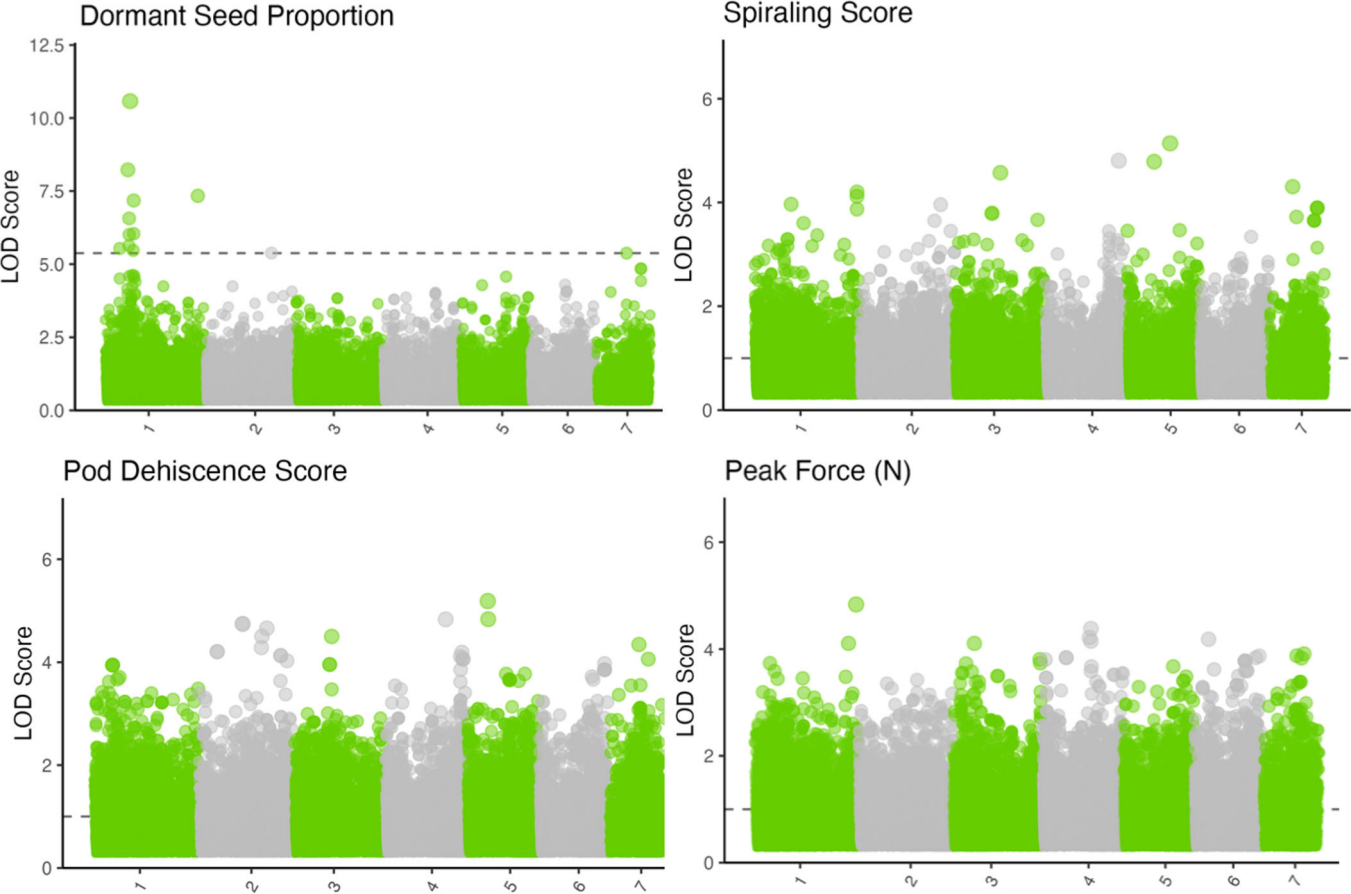
Manhattan plots for physical dormancy proportion, pod spiraling score, pod dehiscence score, and peak force to dehiscence. Colors alternate to differentiate between seven chromosomes. The *y*-axis indicates the logarithm of odds (LOD) score of the *p*-value for each marker model. The horizontal dotted line indicates the *p*-value level equivalent to a false discovery rate of 0.05. If no marker reached the false discovery rate of 0.05, a horizontal line was plotted at LOD 1. Note the unequal *y*-axes.

**Figure 3:**
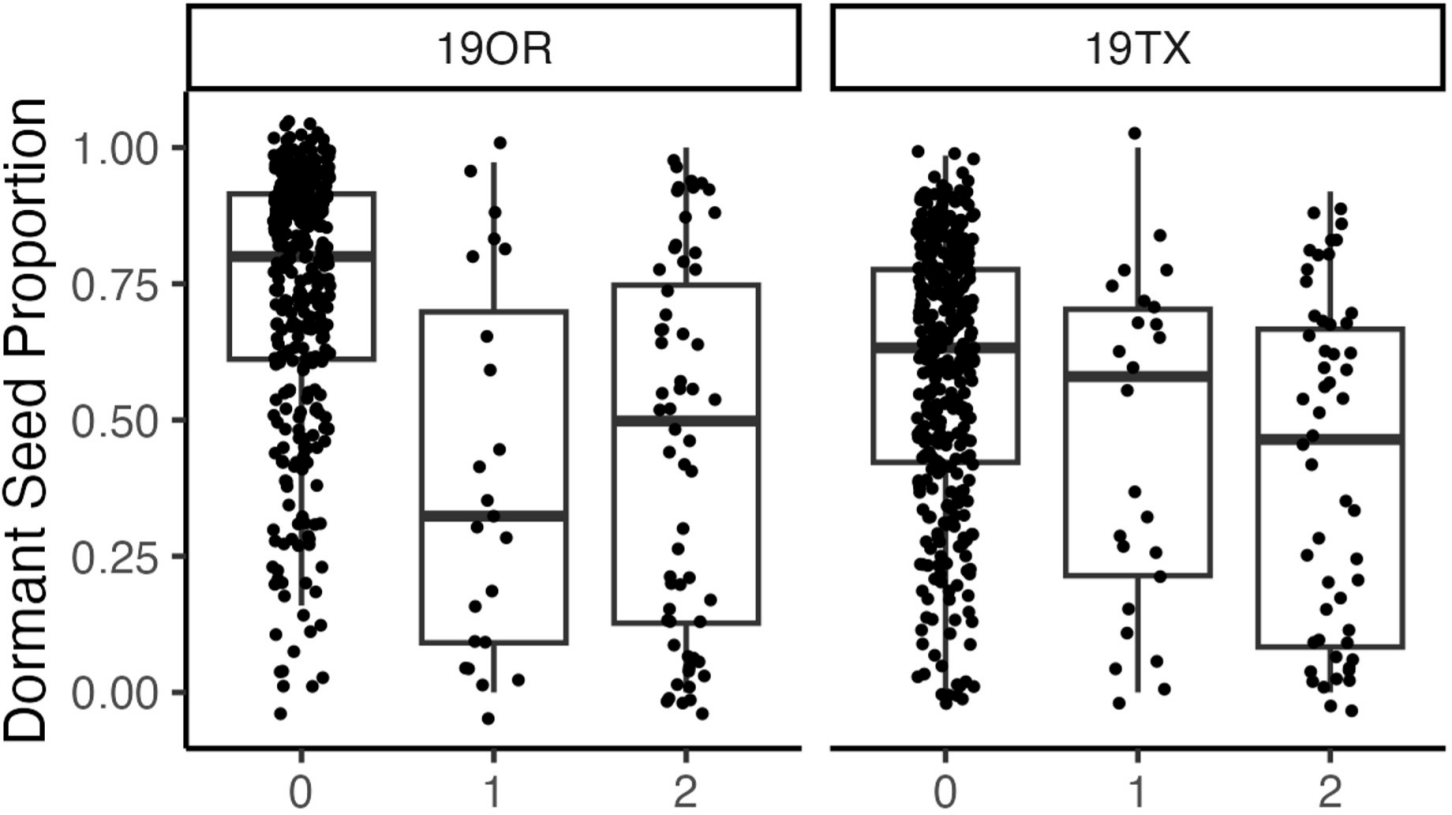
A boxplot indicating the relationship between allele dosage at a major quantitative trait locus (chromosome: 1, position: 63611165) and hard seed proportion at two site years (19OR: Oregon, 19TX: Texas). Each point is the unadjusted hard seed proportion for each individual with each allele dosage.

Seed dormancy BLUPs had multiple significant associations around a primary peak with the lowest *q*-value 1.29 × 10^−5^ (**Figures 2**, **3**; **Supplementary Figure S3**; chromosome: 1, position: 63611165). The reference allele frequency in the population is 0.68, with an estimated additive marker effect of the alternate allele of −0.61 (TX: −0.38 and OR: −0.83). This implausibly large marker effect is due to the strong impact of heterozygotes from Oregon in the dataset (**Figure 3**).

Force to dehiscence BLUPs, spiraling scores, and pod dehiscence score BLUPs had no statistically significant genome-wide associations, with the lowest *q*-value SNP for each trait remaining >0.20. Models run independently for Texas and Oregon sites found similarly insignificant associations within each trait. Furthermore, a multi-trait GWAS model including spiraling scores and pod dehiscence scores did not result in significant QTL despite modest correlations between the traits (**Supplementary Figure S3**; data not presented).

## Discussion

This study found a large effect of QTL on the proportion of dormant seeds in *V. villosa* (**Figure 3**). Assays measuring seed dormancy are labor-intensive (29 min per sample) and cannot be completed prior to pollination of a crossing block. These labor-intensive phenotypic selection cycles have resulted in<10% reduction in seed dormancy per season (data not presented). Therefore, the strong QTL for seed dormancy is useful for reducing labor during breeding cycles and can allow screening prior to flowering. Eliminating seed dormancy will improve *V. villosa* agronomics by reducing volunteers in fields and allowing reduced seeding rates for producers (Kissing Kucek et al., 2020a). Further work is ongoing to (1) find a rapid genotyping platform for marker-assisted selection based on this QTL, (2) validate these QTL in more recent breeding material, and (3) determine potential genes responsible. Further genetic analysis will be aided by recently reported transcripts associated with seed coat formation in *V. villosa* (Ali et al., 2023). Prior reports of legume seed dormancy reduction during domestication are through mutations that weaken the seed coat (Smýkal et al., 2014; Sun et al., 2015; Chai et al., 2016; Sedláková et al., 2021; Soltani et al., 2021).

Given the presence of strong environmental variation, measurement uncertainties, and sequencing errors, the primary seed dormancy QTL could range from having a moderate impact to having complete control over seed dormancy (Beavis, 1998; Kissing Kucek et al., 2020a). Fortunately, there are two independent secondary QTL with *q*-value<0.05 both within (position: 61125989; *q*-value = 0.004) and outside chromosome 1 (chromosome: 7; position: 77125802; *q*-value = 0.046; **Figure 2**; **Supplementary Table S1**). These secondary QTLs have modest statistical support and are independent of each other and the primary QTL (*r* ^2^< 0.2), indicating that they could provide additional or alternative reductions in seed dormancy.

The germplasm in this study has undergone selection for two generations in the Cover Crop Breeding Network. By tracing pedigree information, it was found that the major QTL-reducing seed dormancy was over-represented (>50% beneficial allele frequency) in progeny descended from three germplasm sources: WIHV1, WIHV2, and PI 491408. This is a greater concentration than would occur by chance based on a Chi-squared test for allele counts in WIHV1, WIHV2, and PI 491408 (all *p*< 0.0001). Omitting progeny from these germplasm sources, the allele frequency drops from 32% to 13%. Both WIHV1 and WIHV2 are the results of prior breeding efforts from a diversity panel in Wisconsin. PI 491408 is a collection from China (Inner Mongolia region) from 1984 and has shown generally strong winter survival in Wisconsin. Interestingly, WIHV1 is derived from PI 491408 through its maternal pedigree. These are potential genetic resources for further evaluation and introgression.

Prior studies documenting loci controlling dormant seed have focused on crosses between domesticated and wild-type populations (Forbes and Wells, 1968; Ladizinsky, 1985; Weeden, 2007; Isemura et al., 2010; Isemura et al., 2012; Kongjaimun et al., 2012; Jang et al., 2015). These studies often report that seed dormancy can be controlled by a single or small number of alleles. In contrast, this study reports the presence of one major and multiple minor QTL within the standing variation of a breeding population (**Figure 2**). It is likely there are multiple genetic paths that could eliminate seed dormancy, but only one largely effective QTL would be fixed in a domesticated population. This is probably because the genetic architecture required to maintain seed dormancy (or dehiscence) is polygenic, and any mutation weakening or disabling a required gene could eliminate dormancy. For example, Sun et al. (2015) reported that different *G. max* populations contained distinct mutations reducing seed dormancy, and each induced different seed coat alterations (increased permeability versus increased cracking). Similarly, Soltani et al. (2021) found that a QTL controlling seed water uptake occurred in only 77% of domesticated *Phaseolus vulgaris* L. samples, and that their reported QTL occurred at different rates in different growing environments. The exact mutation that becomes fixed in a cultivated population is likely a mixture of chance and physiological constraints. For example, Chai et al. (2016) reported a loss of seed dormancy in *M. truncatula* through a mutation in the KNOX II gene, but alterations in this gene influenced cutin formation across many organs. This could influence fitness. Other domestication-related mutations appear to have genetic “side-effects” that could be adaptive or maladaptive for a given environment (Weeden, 2007; Soltani et al., 2021). Therefore, semidomesticated populations or meta-populations could segregate for multiple large-effect QTL for domestication traits such as seed dormancy. However, modern cultivars are likely to have only one large effect on QTL because redundant QTL does not provide additional adaptive benefits in agronomic conditions.

No significant QTL were found for pod dehiscence scores, pod spiraling scores, or force to shatter. Interestingly, the surrogate measures for pod dehiscence scores had lower reliability estimates (force to dehiscence: 0.41; spiraling score: 0.42) than the primary phenotype of visual dehiscence scores (0.61). Kissing Kucek et al. (2020b) previously reported force to shatter is a promising trait for phenotyping to differentiate between individuals with low pod dehiscence rates, while spiraling scores are best able to differentiate among individuals with high pod dehiscence rates. In contrast to the extensive time and labor required to phenotype seed dormancy and force-to-dehiscence, pod dehiscence scores can be measured in<1 min per field replicate. Without a significant QTL for dehiscence, visual scoring of pod openness remains a fast method for evaluating dehiscence in breeding programs.

Broadly, the lack of significant pod dehiscence QTL was due to a combination of smaller sample sizes, lower trait reliability, and poor trait distributions at the Oregon site. It is also possible that major QTLs exist in the breeding program but are at a frequency below the statistical detection threshold. Oregon was a much more challenging growing environment for pod shatter traits, with >80% of pod dehiscence scores >2.5 (on a scale of 0–3). Since the force-to-shatter measurement required unshattered pods, this limited the Oregon force-to-shatter sample size to 133. The use of Oregon as a site was strategic since west-central Oregon is a major region for growing cover crop seed. The dry Oregon summers, which enable seed production, will also exacerbate pod dehiscence. Parker et al. (2020) speculated that major new alleles that reduce dehiscence occurred in *P. vulgaris* when introduced to more arid growing conditions. Similarly, continued selection to reduce pod dehiscence in arid seed-producing regions will be required to improve cover crop seed yields.

## Data availability statement

The datasets presented in this study can be found in online repositories. The names of the repository/repositories and accession number(s) can be found below: https://datadryad.org/stash/share/cm3KNBgBAYYOmc4yQlEkMjaVV4mM6s4Eb5LjFNglgH8.

## Author contributions

NT: Formal Analysis, Investigation, Visualization, Writing – original draft. LK: Conceptualization, Data curation, Formal Analysis, Funding acquisition, Investigation, Methodology, Project administration, Resources, Supervision, Visualization, Writing – review & editing. BC: Data curation, Project administration, Supervision, Writing – review & editing. AM: Data curation, Project administration, Supervision, Writing – review & editing. JD: Data curation, Project administration, Supervision, Writing – review & editing. JE: Data curation, Project administration, Supervision, Writing – review & editing. SA: Data curation, Formal Analysis, Investigation, Supervision, Writing – review & editing. JR: Data curation, Supervision, Writing – review & editing. SB: Data curation, Formal Analysis, Writing – review & editing. SM: Data curation, Funding acquisition, Writing – review & editing. MM: Data curation, Project administration, Supervision, Writing – review & editing. SK: Data curation, Writing – review & editing. RH: Data curation, Project administration, Supervision, Writing – review & editing. MA: Data curation, Project administration, Supervision, Writing – review & editing. HR: Conceptualization, Data curation, Funding acquisition, Investigation, Methodology, Project administration, Resources, Supervision, Writing – review & editing.
