# Supplementary material for: Genome-wide association mapping in hairy vetch (Vicia villosa) discovers a large effect locus controlling seed dormancy
Source: Front Plant Sci. 2023 Oct 24;14:1282187. doi: 10.3389/fpls.2023.1282187 (PMC10628312; doi:10.3389/fpls.2023.1282187)
Supplement: Supplementary file 1 [file DataSheet_1.pdf]

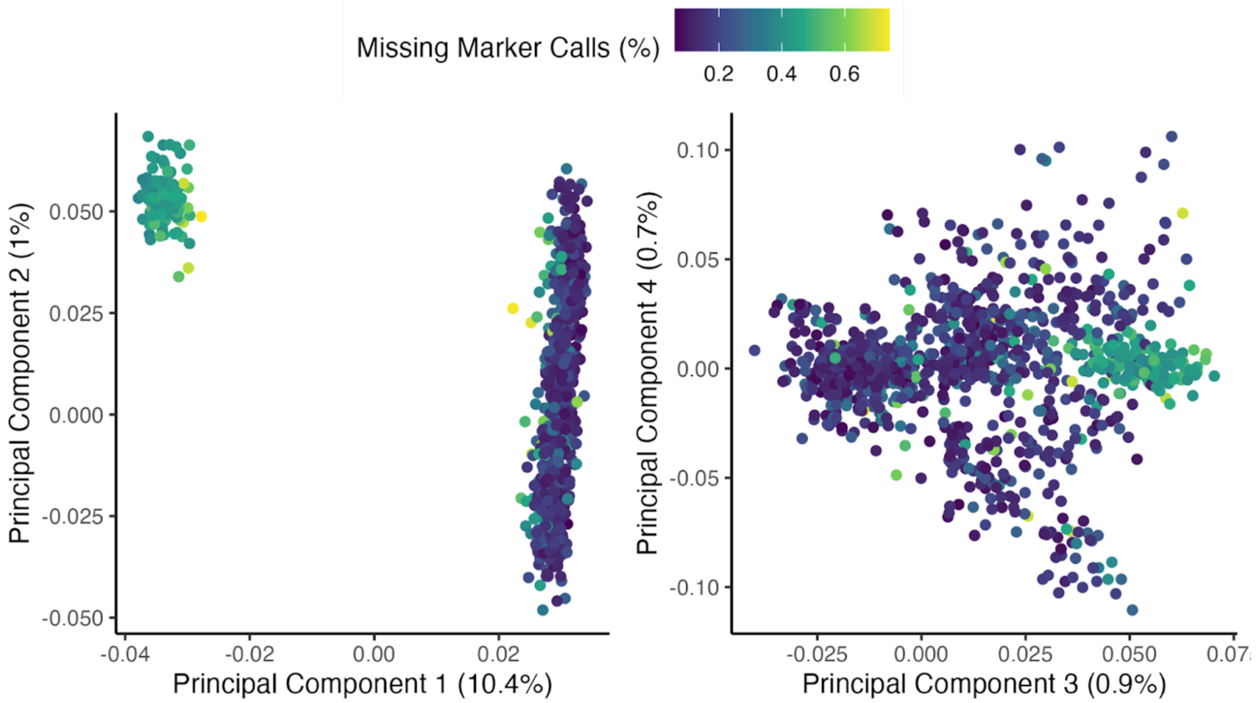

Supplementary Figure 1: The first four principal components of 1,019 *V. villosa* genotypes based on 100,000 randomly selected SNPs. Point colors indicate the proportion of missing markers in each individual. The percent variance of each principal component are indicated in parentheses.

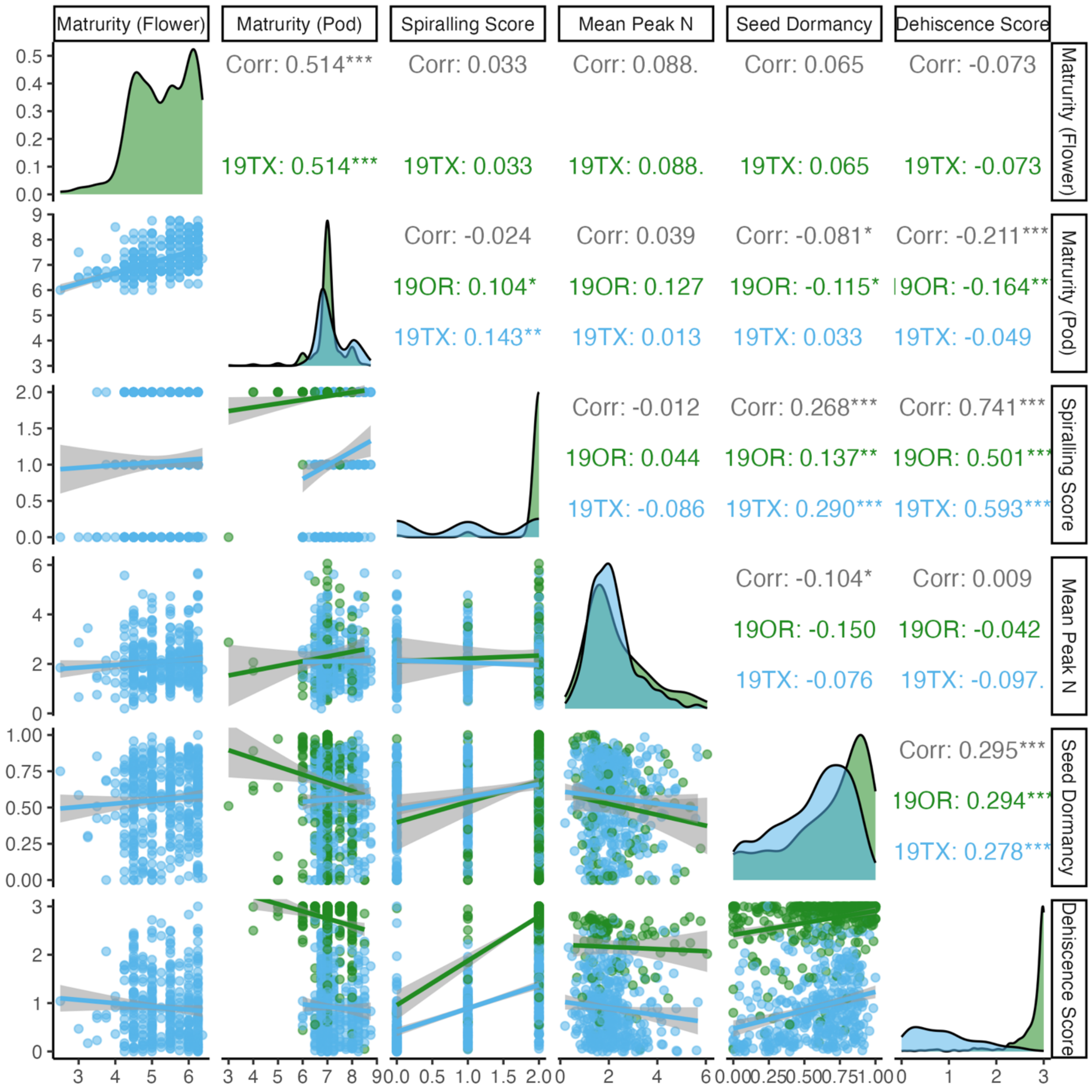

Supplementary Figure 2: Pairwise plots and correlations of maturity scores at flowering (50%), maturity scores at pod development (50%), pod spiraling scores (0,1,2), mean force to shatter (peak N), seed dormancy, and mean dehiscence scores. Point clouds (below diagonal), histograms (diagonal), and correlations (above diagonal) are divided by site-year with Oregon in green and Texas in blue.

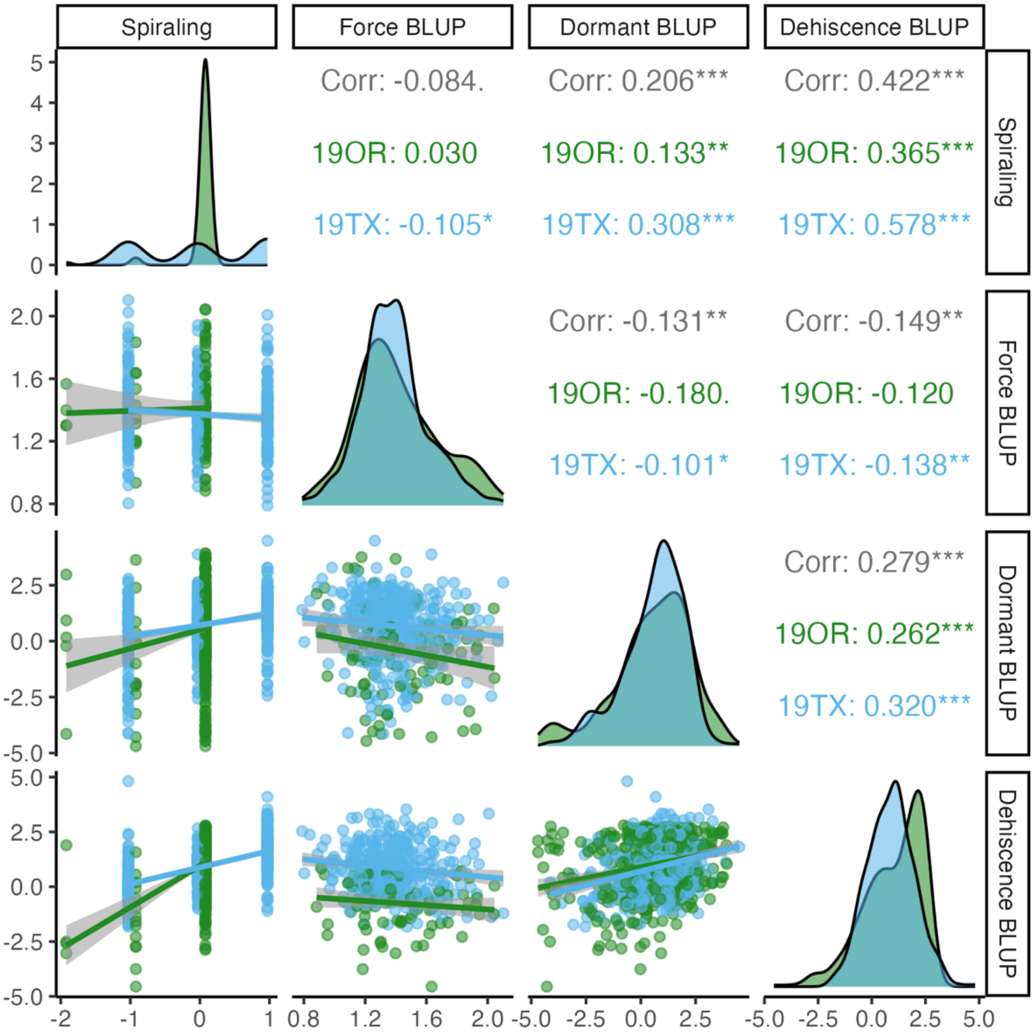

Supplementary Figure 3: Pairwise plots and correlations of pod spiraling scores (0, 1, 2), force BLUP, dormancy (hard seed) BLUP, and pod dehiscence (shatter) BLUP. Point clouds (below diagonal), histograms (diagonal), and correlations (above diagonal) are divided by site-year with Oregon in green and Texas in blue.

Chr	Position	LOD Score (p-value)	q-value	Ref	Alt	Allele Frequency	Effect	Texas Effect	Oregon Effect	Correlation to Chr 1: 63611165
1	63611165	10	1.29×10^{-05}	T	A	0.32	-0.61+-0.09	-0.38	-0.83	1.00
1	57882973	9	0.00024	A	T	0.22	-0.60+-0.09	-0.45	-0.73	0.57
1	61125989	7	0.00487	T	G	0.68	-0.45+-0.08	-0.41	-0.48	0.17
1	72506717	7	0.00487	T	C	0.28	-0.46+-0.09	-0.33	-0.58	0.54
1	234565325	7	0.00487	G	A	0.19	-0.59+-0.10	-0.37	-0.76	0.41
1	61392318	7	0.01860	T	C	0.72	-0.36+-0.07	-0.32	-0.4	0.23
1	72506748	6	0.04321	A	T	0.29	-0.42+-0.09	-0.28	-0.55	0.56
7	77125802	6	0.04688	T	G	0.49	-0.30+-0.07	-0.32	-0.28	0.06
1	71381149	6	0.05173	T	C	0.19	-0.47+-0.11	-0.4	-0.53	0.38
1	73986364	6	0.09604	T	C	0.92	0.26+-0.06	0.29	0.22	-0.07

Supplementary Table 1: Candidate SNP markers with q-values <0.10 in a model predicting the proportion of dormant seed from a *V. villosa* individual. Q-values are based on the false discovery rate. Marker effects are estimated as fixed effects in models otherwise identical to the initial GWAS analysis.

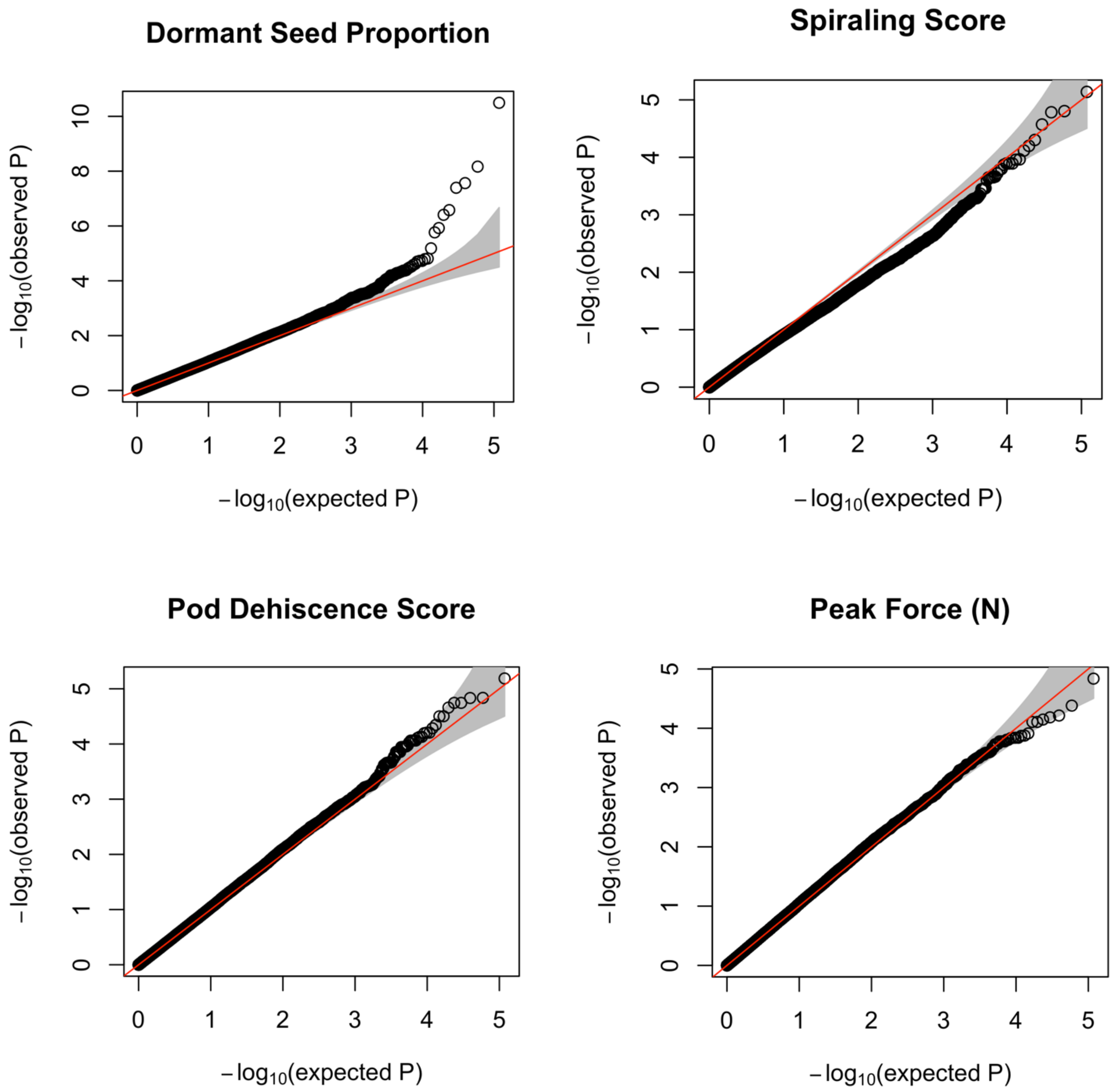

Supplementary Figure 4: Quantile-quantile plots of expected and observed p-values for association tests of dormancy (hard seed) proportion, spiraling score, dehiscence (pod shatter), pod shatter score, and peak force.
